# Exploring and Developing the Workplace Health Culture Scale in Taiwan

**DOI:** 10.3389/fpubh.2019.00397

**Published:** 2020-01-10

**Authors:** Yao-Tsung Chang, Feng-Jen Tsai, Chien-Chih Kuo, Ching-Ying Yeh, Ruey-Yu Chen

**Affiliations:** ^1^School of Public Health, Taipei Medical University, Taipei, Taiwan; ^2^PhD Program in Global Health and Health Security, and Master Program in Global Health and Development, College of Public Health, Taipei Medical University, Taipei, Taiwan; ^3^Department of Psychology, National Cheng-Chi University, Taipei, Taiwan

**Keywords:** workplace health culture, scale, factor analysis, workplace health promotion, health behavior

## Abstract

**Background:** The aim of this study was to develop and validate the workplace health culture scale.

**Methods:** This paper collected and re-organized current definitions about health culture from literature and created the domains and items to develop a new tool. Six enterprises and 2,431 participants were recruited from northern Taiwan for validity test.

**Results:** We found the workplace health culture scale had appropriate reliability and validity, including a good model fit for the 25-item scale.

**Conclusions:** Workplace health culture might be an important domain to the work of WHP. More validity and reliability studies about WHCS in wider industries and the correlation between WHCS and other WHP indicators are needed.

## Background

Many chronic diseases like cardiovascular diseases and type 2 diabetes are associated with obesity. Most of these are caused by the increasing sedentary lifestyles, unhealthy diets, and many other facets of an unhealthy lifestyle (1, 2). Since most adults have over half of their waking time working in the workplaces, to promote people's healthy behaviors and their health conditions, worksite health promotion (WHP) has become necessary. Over the past three decades, many WHP studies have focused on how to implement effective intervention and measure the effectiveness of WHP and the cost-effectiveness of WHP programs. However, the overweight and obesity rates are still going up all over the world (3), and the evidence of the effectiveness of WHP program is also inconsistent. To clarify the factors which influence the successfulness of WHP program, more studies relating to the health behaviors, barriers, promoters, and supporting environment were created. In recent years, there is one issue having received attention gradually: Workplace health culture.

Culture is an abstract concept which describes employees' attitudes and behaviors, and norms that is very ethnically and geographically specific, and it will affect specific behaviors (4, 5). Organizational culture is an important element of business management and there are kinds of cultures in different dimensions, including safety culture and healthy culture. A related concept is the “worksite culture of health” which includes the gamut of organizational factors that work to encourage healthy lifestyle choices (6–9). Currently, safety culture is a mature dimension and has received attention earlier than health culture (10–13). Many companies have created their safety culture to keep employees' safe, reduce the costs associated with work-related accident, and enhance their corporate image. As the comprehensive occupational safety program can benefit the work of preventing injury, successful workplace wellness programs must be tailored to employees' health needs and wishes as well as complementing each organization's unique culture (9, 14). For healthy behaviors, culture acts as an interpersonal force to increase or decrease motivation like self-determination and self-efficacy (15, 16). This means that just as the safety culture and employee safety are related and important, if we want to effectively promote the healthy lifestyle of employees, healthy culture is inevitably a very important issue of WHP intervention, and it is necessary creating workplace health culture for implementing more comprehensive and better effectiveness worksite health promotion in the future.

The health culture concept is understood to be a set of core attributes engendered by the interaction of social and organizational systems that reflect the values, assumptions, expectations, and definitions of workers that in turn affect the way workers think, feel, and behave with regard to personal and group health (17, 18). Until now, there are not many studies on health culture, and only a few tools are available to measure this construct; at the same time, all of them have different definitions of the components of health culture (7, 19–22). Although we are not sure whether the existing health culture measurement tools are applicable to Taiwan, some items related to local regulations or habits may reduce the applicability of these tools, and some questions do not apply to Taiwan's health culture assessment. Therefore, the development of a more extensive tool can not only help Taiwan's work of WHP, but also contribute to the promotion of this study issue. The purpose of this study was to develop and validate Workplace Health Culture Scale (WHCS) to improve the work of WHP in Taiwan.

## Methods

This study focused on developing and validating the WHCS between August 2017 and June 2019, and it was approved by the Institutional Review Board (IRB) of Taipei Medical University and budget supplements were granted by the Ministry of Science and Technology Taiwan. In this study, three phases were carried out to develop WHCS: (1) reviewing literature and defining domain, (2) item generation, (3) validation test. Data were collected from six companies in Northern Taiwan between June 2018 and December 2018.

### Development of Workplace Health Scale and the Definition of Domains

First, we organized an expert group to integrate literature and define domains. The expert group consisted of seven health and psychology professionals, including one WHP and health behavior expert, one WHP and occupational safety and health (OHS) expert, one Occupational Safety and Health Administration supervisor, one workplace health productivity expert, one global health expert, one industrial and organizational psychologist, and one statistician who has experience in scale development. In addition, the industrial and organizational psychologist has a good experience in developing the safety culture scale (23–25). In the first session, we integrated the definition and framework of workplace health culture which was mainly collected from Allen (6), Aldana (26), and Kent's (18) study. Table 1 contains a list of our domain definitions. According to the definition of culture, the expert group believed the WHCS items would need to focus on employees' cognition, attitudes, and feelings toward workplace health promotion. Therefore, we generated eight domains as follows: (1) Supporting Environment, (2) Health Policy, (3) Health Climate, (4) Peer Support, (5) Supervisor Support and Role Modeling, (6) Health Involvement, (7) Personal Value, and (8) Common Value.

**Table 1:** Definitions of the workplace health culture scale (first edition).

**Domains**	**Definition**
Supporting environment	The feelings and attitudes about the physical environment of sports, diets, and psychological health, and the importance of the company's emphasis on the employees' health promotion issues
Health policy	Employees' attitudes toward company policies on health promotion, including physical activities, healthy diets, psychological health, and work-life balance
Health climate	Employees' attitudes and feelings toward health promotion within the company
Peer support	Employees' feelings and attitudes about encouraging each other to have a healthy lifestyle and even forming groups to promote health
Supervisor support and role modeling	Employees' feelings about supervisors' attitudes toward health promotion, and the extent to which the supervisors play the role models
Health involvement	Employees' attitudes, behaviors, and responsibilities on the WHP activities they participate in
Personal value	Individual's beliefs, attitudes, and cognition toward health promotion
Common value	The common beliefs and attitudes toward health promotion

Then we generated items to these domains with a total of 67 items. Every item began with the heading “I think…” or “My colleagues and I feel that…” to reflect the employees' attitudes and feelings. We recruited additional two WHP and OHS experts to join the expert group and to check the feasibility and content validity based on the five-point Likert scale and open-ended feedback. Items remained on the list were according to the four criteria: (1) fitness ≧ 3.0 points, (2) importance ≧ 3.0 points, (3) description clarity ≧ 3.0 points, and (4) experts' specific amendments.

Finally, there were 65 items remaining on the list with descriptions on some items being corrected. The supporting environment contained six items, including the physical activity/healthy diet/psychological health/health risk assessment/health management system environment or service that made employees feel health was valued by the employer; the health policy contained 10 items, including the attitude of whether the health policies could be effectively implemented and carried out; the health climate contained 10 items, including five items on which the employees felt about a specific health climate in the company (e.g., physical activity climate), and five items on which the employees felt about the supervisor's attitudes of a specific healthy lifestyle and health behavior; the peer support contained eight items which looked at the employees' feelings and attitudes about encouraging each other to have a healthy lifestyle; the supervisor support and role modeling contained nine items, including two items about the CEO's healthy lifestyle role modeling, two items about the direct supervisors' role modeling, and five items about the direct supervisors' support on healthy lifestyle; the health involvement contained eight items which looked at the employees' attitudes on the healthy activities they participated in; the personal value contained five items which examined employees' personal beliefs and attitudes toward WHP; and the common value contained 10 items which looked into the employees' common beliefs and attitudes toward health promotion. Considering the original survey items are constructed in Chinese language with the rigor of translation in the context, for more detailed information about specific items, please contact the author(s).

After the completion of the WHCS first edition, we started inviting companies for validity test. Considering the need of representative and diversified samples, we recruited six companies from three different industries and company sizes. Construct validity and composite reliability were assessed to verify our health culture framework and to determine the appropriateness of items by both exploratory factor analysis and confirmatory factor analysis. We also tested the discriminant validity among companies and the internal consistency of the WHCS's final version.

### Samples

Six companies were recruited from Northern Taiwan, including the large-sized bank A, the small-sized bank B, the large-sized manufacturers C and D, the large-sized technology company E, and the medium-sized retail and wholesale company F. Engagement in WHP varied among the companies (e.g., in terms of the issues they address, level of comprehensiveness, and maturity of program). Three companies were awarded the 2018 Health Promotion Administration Ministry of Health and Welfare's WHP prize (Companies C, E, and F), which is the highest official certification and honorary award a company can earn for workplace health promotion in Taiwan, and it has been in operation for 12 years. In this study, the participating enterprises were recruited from a pool of the certified companies. We invited and collected samples from each of the small and medium-sized enterprises, aiming for at least 50% participating rate. Considering a greater number of employees in the technology company E (~6,000 employees) than other companies in this study, we collected 1,000 samples from the company E to ensure an adequate representation. Between June and December 2018, we sent online questionnaire to the target companies and had the assistance from the health promotion leaders of these companies in the promotion and recruitment, and participants were rewarded 50 New Taiwan dollars for each questionnaire. Out of the 2,575 total survey respondents, 2,431 completed the questionnaire. This represented ~16.4–97.8% of the eligible employee population, with small-size company (B) having relatively higher participation rates and the large-size company (E) the lowest. We did not separate domains in this survey but combined all the 65 items into one questionnaire.

### Statistical Analysis

The validation test included exploratory factor analysis and confirmatory factor analysis. Before the factor analysis, we adjusted our sample size. Considering the recommendations of sample size for conducting factor analysis (27), we decided to use 10 times the number of questions for exploratory factor analysis and confirmatory factor analysis. We randomly extracted 300 samples from company E and combined with other samples, and then we randomly extracted 650 samples from this large sample pool (*n* = 2,431) for analysis. We confirmed that the randomly extracted samples were not significantly different from the original sample demographic characteristics.

The exploratory factor analysis was used for reducing the number of items. We used the principal component factor analysis with the varimax rotation and eigenvalue criterion >1.0 to detect the latent variable. Before analysis, we confirmed the feasibility of factor analysis by Kaiser-Meyer-Olkin test (KMO) and Bartlett's test of sphericity. Then, items with factor loading < 0.50, cross-loading > 0.40, or communalities < 0.30 were eliminated (28, 29). In addition, every latent variable had to have at least three factors. The exploratory factor analysis was analyzed at a 95% significance level and conducted using PASW 22.0 software for Windows (SPSS, Chicago, IL).

After the factor analysis, we performed the confirmatory factor analysis to build the conceptual model and compared it with the original model (as Table 1 list) to establish construct validity and reliability of WHCS. Before full model building, every latent variable collected from confirmatory factor analysis was tested separately for the model fitness to detect any unsuitable item. The following criteria of model fitness were used: root mean square error of approximation (RMSEA) < 0.08 (and 0.05 or lower should be better), χ^2^/df < 5, standardized root mean square residual (SRMR) <0.08, comparative fit index (CFI) > 0.90, Tucker-Lewis index (TLI) > 0.90 (30–33). In addition, the composite reliability (CR) needed to be >0.7 for appropriate construct reliability (34), and average variance extracted (AVE) had to be >0.5 for appropriate convergent validity (35). We used AMOS 20.0 software to conduct confirmatory factor analysis (Chicago, IL).

Finally, we conducted ANOVA test for the six companies with the final version of WHSC to detect the discriminant validity of different degrees of WHP and the companies. The Cronbach's α was also tested for appropriate content reliability (36), and the threshold was 0.70 or greater. The test was analyzed at a 95% significance level (*P* < 0.05).

## Results

### Demographics

The demographic characteristics of the study samples (*N* = 2,431) are listed in Table 2. The total number of workers of these six companies ranged from 188 to 6,270. The two banks had significantly higher proportion of female workers. The technology company had the highest proportion of employees with a master's degree or higher education level (59.7%), the lowest average age (37.7 ± 9.68 years), and the lowest average seniority (5.5 ± 4.55 years). In general, most of the samples had an educational level with at least a bachelor's degree.

**Table 2:** Demographic characteristics of samples.

	**Companies** ***N* (%)**					
	**Bank A** **(*n* = 267)**	**Bank B** **(*n* = 139)**	**Manufactory C** **(*n* = 264)**	**Manufactory D** **(*n* = 328)**	**Technology company E** **(*n* = 1,029)**	**Wholesale retailer F** **(*n* =322)**
**Gender**						
Male	128 (47.9)	47 (33.8)	206 (78.0)	166 (50.6)	637 (61.9)	207 (64.3)
Female	139 (52.1)	92 (66.2)	58 (22.0)	162 (49.4)	392 (38.1)	115 (35.7)
**Educational level**						
Lower than junior high school	0 (0.0)	3 (2.2)	1 (0.3)	10 (3.1)	2 (0.2)	0 (0.0)
Senior high school	11 (4.2)	15 (10.9)	53 (20.2)	40 (12.2)	6 (0.6)	35 (11.0)
University	194 (74.3)	68 (49.7)	174 (66.2)	210 (64.2)	407 (39.6)	222 (70.1)
Master's degree or higher	56 (21.5)	51 (37.2)	35 (13.3)	67 (20.5)	614 (59.6)	60 (18.9)
**Age, years**						
18–29	43 (16.4)	14 (10.5)	23 (8.7)	30 (9.2)	368 (35.7)	30 (9.5)
30–39	93 (35.5)	49 (36.8)	65 (24.7)	110 (33.8)	489 (47.5)	136 (43.0)
40–49	83 (31.7)	21 (15.8)	86 (32.8)	96 (29.4)	159 (15.5)	105 (33.3)
50–64	40 (15.3)	48 (36.1)	89 (33.8)	89 (27.3)	13 (1.3)	45 (14.2)
65 or higher	3 (1.1)	1 (0.8)	0 (0.0)	1 (0.3)	0 (0.0)	0 (0.0)
Age, years (Mean ± SD)	39.6 ± 10.13	42.5 ± 12.49	43.6 ± 9.64	42.6 ± 10.19	37.7 ± 9.68	39.8 ± 8.45
Seniority, years (Mean ± SD)	13.2 ± 10.14	13.1 ± 11.82	18.1 ± 9.81	15.2 ± 11.48	5.5 ± 4.55	8.9 ± 7.15
Total number of workers	923	188	3,229	1,204	6,270	323

*SD, standard deviation*.

### Exploratory Factor Analysis

The first exploratory factor analysis produced 11 latent variables, accounting for 68.57% total variance. The KMO test of sampling adequacy was 0.967 and Bartlett's test for sphericity was highly significant (*p* < 0.001). The original 65 items were gradually eliminated according to their factor loading and cross-loading criteria, and eventually 38 items remained. There were 27 items eliminated as follows: 11 items had factor loading lower than 0.50, 12 items had cross-factor loading higher than 0.40, and two latent variables had only two items of each (and only <2% total explained variance). The two eliminated latent variables were separated from the “Supporting Environment” domain and the “Peer Support” domain. The factor analysis of the rest of the 38 items produced seven latent variables, accounting for 63.19% total variance. Most of the items remained in the original domains except Q15, which was classified as “Health climate” from “Health policy.” Therefore, we did not change any domain's label.

### Confirmatory Factor Analysis

The confirmatory factor analysis tested each of the seven domains after the exploratory factor analysis test. However, only four domains could be analyzed since the other three domains had only three items each (just-identified). The first domain—“Health Climate”—which had the highest total explained variance in exploratory factor analysis had 11 items. We eliminated Q15, Q17, Q19, and Q24 since they had higher modification index (M.I.) value and high significant correlation with other items. The second domain—“Common Value”—had seven items. We eliminated Q58 for the same reason. The third domain—“Supervisor Support and Role Modeling”—had five items, and we eliminated Q35 since it significant correlated to Q37 and Q39, and it had a high M.I. value. The fourth domain—“Supporting Environment”—had four items, and it was the only domain that was eliminated because Q1 and Q3, Q2 and Q4 had very high significant correlation with each other that lacked enough model fit. The fifth domain—“Health Policy”—had five items. We eliminated Q9 due to its high M.I. value.

Six domains were constructed from the remaining 25 items, and the total explained variance was 69.64% (Table 3). All of the domains could meet the criteria of CR and AVE, and the Cronbach's α was 0.804–0.919. Table 4 showed the model fit and the correlations among the domains. The two domains—“Health Climate” and “Supervisor Support and Role Modeling”—had worse model fit than others since each item within the domains had a certain degree of correlation (Health Climate had RMSEA = 0.075, χ^2^/df = 4.879, Supervisor Support and Role Modeling had RMSEA = 0.079, χ^2^/df = 5.275). The full model exhibited enough fit statistics (RMSEA = 0.059, χ^2^/df = 3.412, SRMR = 0.060, CFI = 0.952, GFI = 0.917, TLI = 0.938). The model fit did not have significant improvement when we eliminated some items with a relatively high M.I. value in the full model (e.g., Q16 and Q42); therefore, Q16 and Q42 were retained. Comparing to our original domain definition, two domains were eliminated (Supporting Environment and Health Involvement), but most items could be correctly classified under the original domain, hence we believe that this scale has sufficient construct validity. All of the domains' Cronbach's alpha were above 0.90.

**Table 3:** Factor loadings for 25-items workplace health culture scale (*N* = 650).

**Domain**	**Original item code**	**Cronbach's α**	**Corrected item-to-total correlation**	**Factor loadings**						**CR**	**AVE**	**Total explained variance (%)**
				**1**	**2**	**3**	**4**	**5**	**6**			
Health climate	Q16	0.919	0.687	0.698						0.92	0.63	18.94
	Q18		0.724	0.693								
	Q20		0.795	0.756								
	Q21		0.770	0.775								
	Q22		0.809	0.758								
	Q23		0.739	0.667								
	Q25		0.733	0.682								
Common value	Q57	0.880	0.701		0.711					0.88	0.60	14.32
	Q59		0.789		0.827							
	Q60		0.763		0.810							
	Q61		0.639		0.727							
	Q64		0.677		0.765							
Supervisor support and role modeling	Q37	0.899	0.707			0.688				0.91	0.71	12.31
	Q38		0.842			0.838						
	Q39		0.830			0.818						
	Q42		0.728			0.728						
Personal value	Q51	0.918	0.831				0.814			0.92	0.79	10.46
	Q52		0.872				0.869					
	Q53		0.801				0.836					
Health policy	Q6	0.809	0.607					0.635		0.82	0.61	8.97
	Q7		0.748					0.825				
	Q8		0.627					0.797				
Peer support	Q26	0.804	0.571						0.612	0.82	0.60	8.58
	Q32		0.688						0.800			
	Q33		0.706						0.774			

*CR, composite reliability; AVE, average variance extracted*.

**Table 4:** Corrections among constructs.

**Domain**	**Model fit**				**Correlations**					
	**RMSEA**	**χ^2^/df**	**SRMR**	**CFI**						
Health climate	0.075	4.879	0.023	0.982	–					
Common value	0.048	2.611	0.014	0.995	0.458^**^	–				
Supervisor support and role modeling	0.079	5.275	0.016	0.995	0.637^**^	0.474^**^	–			
Personal value	–	–	–	–	0.499^**^	0.479^**^	0.514^**^	–		
Health policy	–	–	–	–	0.641^**^	0.306^**^	0.403^**^	0.347^**^	–	
Peer support	–	–	–	–	0.585^**^	0.512^**^	0.552^**^	0.433^**^	0.431^**^	–
Full model	0.059	3.412	0.060							

*RMSEA, root mean square error of approximation; SRMR, standardized root mean square residual; CFI, comparative fit index*.

** *p < 0.01*.

The final model had good discriminant validity (Table 5). All of the domains and the total score had significant differences among the 6 companies. In general, those companies which won the WHP prize in 2018 (Company C, E, and F) had significant higher points than others, but the small bank B had the highest peer support point. Therefore, the final version of WHCS has six domains–Health Climate, Common Value, Supervisor Support and Role Modeling, Peer Support, Personal Value and Common Value–and 25 items in total.

**Table 5:** The result of discriminant validity test.

	**Companies** **(Mean ± SD)**						
	**Bank A** **(*n* = 262)**	**Bank B** **(*n* = 134)**	**Manufactory C** **(*n* = 263)**	**Manufactory D** **(*n* = 326)**	**Technology company E** **(*n* = 1029)**	**Wholesale retailer F** **(*n* = 316)**	***p* value**
Health climate	21.3 ± 5.70	24.8 ± 4.69	24.9 ± 4.79	23.2 ± 4.76	24.3 ± 4.49	27.2 ± 4.16	<0.001^**^
Common value	19.9 ± 2.61	21.1 ± 2.54	21.0 ± 2.19	20.5 ± 2.36	20.5 ± 2.59	20.8 ± 2.56	<0.001^**^
Supervisor support and role modeling	13.3 ± 3.22	14.5 ± 3.04	14.6 ± 2.53	14.4 ± 2.64	14.8 ± 2.74	15.3 ± 2.68	<0.001^**^
Personal value	10.4 ± 2.23	10.9 ± 2.16	11.3 ± 1.93	10.7 ± 1.88	10.9 ± 2.08	11.2 ± 2.05	<0.001^**^
Health policy	8.1 ± 4.03	8.9 ± 3.66	10.0 ± 3.24	8.2 ± 4.12	9.3 ± 3.22	11.0 ± 2.92	<0.001^**^
Peer support	10.5 ± 2.07	12.1 ± 1.51	11.2 ± 1.86	11.0 ± 1.91	11.2 ± 1.99	11.3 ± 1.78	<0.001^**^
Total score	83.5 ± 14.6	92.4 ± 13.4	93.1 ± 12.90	88.1 ± 12.67	91.1 ± 12.68	96.7 ± 12.49	<0.001^**^

*SD, standard deviation*.

** *p < 0.01*.

## Discussion

This is the first Chinese workplace health culture audit with appropriate development and validation work. In this study, we developed and validated the WHCS, and it had appropriate construct validity, content validity, discriminant validity, composite reliability, and internal consistency. This scale was developed based on several current workplace health culture and audit literature, and we believe it can appropriately reflect the workplace health culture and can be used for the improvement of WHP.

Considering our goal was to measure the workplace health culture and none of the existing tools could truly meet our needs in addressing the construct of interest, we saw the need of creating a new tool which is the frame and design of the “culture” definition from our point of view and the quality of the existing tools. Before we developed WHCS, we compared all the current health culture measurement tools (6, 7, 18, 20, 21) and took all the current health culture frame and theory into consideration (17, 22, 26, 37, 38). Health culture is a collective cognition and attitude, which is built on the individual's subjective and abstractive attitude, values, and beliefs to health. However, some of current health tools do not meet our definition, e.g., Jia's Chinese workplace health culture scale (21) and Kent's Culture of health scale (38). Jia's Chinese Workplace Health Culture scale had a lot of items related to direct supervisor's health behaviors (e.g., smoking, drinking, exercise) and the support and encouragement from the direct supervisor and family to lead a healthy lifestyle; it was not about the workplace health culture but the employees' cognition and attitude. Kent's Culture of health scale consisted of two parts: internal and external culture of health, and this scale was more likely a culture checklist rather than a personal questionnaire. For example, it asked the number of employees who were given health education in the past 12 months, whether the organization had a health promotion strategic plan, and it contained some open-ended questions to explore the organizational commitment and volunteerism. Aldana's review study pointed out that several health culture measurement tools developed before 2012 had some shortcomings, which were verified to be less relevant to health (19) or lacked evidence of reliability and validity (39). In addition, we also excluded those scales related to “health climate” since it was not the same as culture. Organizational culture and climate are not the same thing (40), climate has narrower concept than culture; however, both of them focus on the shared perception, values, and beliefs rather than the actual environment evaluation. It means the health culture measurement is not the evaluation of the degree of the accessibility or applicability in the health policy and physical and psychological environment, nor is it about the feeling of employees toward them. Finally, after checking the LHCA and Kwon's Culture of Health scale (COH) and considering that the translation and modification of the questionnaire would result in subtle variation in the semantics and that some questions might be irrelevant to the regulations in Taiwan, we finally decided to develop a new scale.

In general, WHCS has six domains and 25 items. Comparing to our original domain definition, only two domains were eliminated, and most items could be classified under the original domain. It might mean that our item design was appropriate and close to the original definition. In WHCS, we put more emphasis on the concept of collective cognition and attitude, so we excluded the investigation about the physical normative and physical environment. Comparing to Allen's Lifegain framework and Lifegain Health Culture Audit (LHCA) (6, 7), WHCS does not include the “normative” domain, and the domain of values is divided into personal and common values. In addition, the supporting environment and health policy domain also focus on “whether these supporting material works” instead of “the availability of materials,” and it is the most obvious difference from the LHCA. Comparing to COH (41), which separates the definition of the supporting environment into two parts—“environment components” and “culture components,” our domains and items are closer to the “culture components” part. Considering the “organizational culture” was defined as a kind of “shared basic assumptions” (37), the workplace health culture should have similar definitions and characteristics. In general, since the definitions of health culture are inconsistent until now, the criterion validity might not be appropriate to the development of workplace health culture tools.

The exclusion of the domain about support environment may be quite reasonable, and it's not just because the model fits problem. In addition to the environment evaluation might not close to the culture definition, there might be another explanation. Until now, there have been many intervention studies in WHP that adopted supporting environment interventions alone or combined with other intervention, but the evidence of effectiveness is still inconsistent. Some studies have found that adopting environmental interventions alone is completely ineffective in improving employees' health behaviors and health status (42–45). However, combined with other behavioral interventions, environmental intervention can help to promote the effectiveness of WHP (46, 47). Therefore, most countries now encourage comprehensive WHP as much as possible. Such research evidence may represent that the supporting environment's influence on changing health attitudes or behaviors is more indirect than other domain of health culture or direct work of behavioral intervention, and this might explain why this domain was excluded in this study.

Another domain excluded in this study is “Health Involvement.” Health involvement was related to employees' attitudes, behaviors, and responsibilities they had participating in the WHP activities, and we assumed it might be affected by the quality and comprehensiveness of WHP, which were different among the six workplaces. It might cause the cyclical interference of health involvement and its impact factor. For example, there were more companies believing that employees' health was entirely their own responsibility, especially for the small and medium-sized enterprises (48, 49), and it would affect employees' attitudes and health involvement in participation (50, 51). Although it was more complicated than other domains and was excluded from the WHCS, we believe that it is still very important and there should be more in-depth research with regard to worksite health culture.

Although we excluded the investigation of physical environment of WHCS, it might be an interesting issue to be studied in the future. In this study, we noticed the health culture might not directly associate with the business size (Table 5). The small and midsized business might have lower resources to WHP program; however, recent studies have shown that the effectiveness of WHP does not entirely depend on the size of the company (52). For example, small businesses might be more likely to shape the peer support in health. On the contrary, large enterprises might not necessarily create a better health culture even if they have the ability to invest more physical materials and resources in the WHP. The question “What kind of WHP investment might shape the health culture more effectively?,” or “Which work of WHP might evoke a positive attitude and cognition to health and build supporting climate for each other?” (e.g., how long, to whom, and to what degree the comprehensiveness of the WHP intervention is suitable to change the culture?) might be the most important field to the WHP in the future.

Our study has several strengths. First, we conducted a complete review of the definition and related literature on organizational culture and health culture and compared existing measurement tools. It seems that not all of the health culture measurement tools are in compliance with our definition of culture nor can they provide us with suitable measurement. Our second strength was that we filled in the gap that only few tools had enough comprehensive structure. This strength might increase the applicability of the tool outside of Taiwan since most of the items focus on the subjective attitudes and employees' cognition and less on objective policies and environment at the substantive level, but we still believe that this requires rigorous verification and testing. In addition, though the final version of WHC consists of only 25 items, it is still good model fit that we believe it yielded sound response in this study.

The limitations of this study could be used to build future research. First, an effective, validated representation of WHCS should incorporate wider varieties of industries across different sizes of enterprises, and it should also include confirmatory factor analysis and model fit tests. Second, we would take into consideration of assigning different weight to each domain of WHCS in the future since it has an unbalanced number of items in each domain. Therefore, our next study will focus on the extensive validation of this tool and its relevance to the environment, employees' health behaviors, demographic characteristics and other factors that may influence health culture, and the association to the health risk.

## Conclusions

In this study, we developed and validated the WHCS, and the results of this study indicated the WHCS has appropriate reliability and validity. WHCS is suitable for measuring employees' attitudes, cognition, and feelings of a healthy workplace for improving the WHP in Taiwan. Based on the limitations and strengths of this study, we suggest that more studies about reliability and validity of WHCS and the correlation between WHCS and other WHP measuring indicators (e.g., personal health behavior and physical and psychological environment) are needed.

## Data Availability Statement

The raw data supporting the conclusions of this manuscript will be made available by the authors, without undue reservation, to any qualified researcher.

## Ethics Statement

Taipei Medical University- Joint Institutional Review Board Committee waived the requirement for written informed consent for participants in this study due to the questionnaire for this study was completely anonymous and de-linked, and the written informed consent was the only source that could link to the respondent's individual. In addition, the questionnaire is distributed by the research team, or the online questionnaire is used, and the content of the questionnaire has the minimum personal risk, in accordance with the national legislation and the institutional requirements.

## Author Contributions

Y-TC, R-YC, F-JT, C-CK, and C-YY participated in the conception, design of the study, and scale development. Y-TC participated in acquisition of data. Y-TC, R-YC, and C-YY performed the statistical analysis and drafted the manuscript. All authors read and approved the final manuscript.

### Conflict of Interest

The authors declare that the research was conducted in the absence of any commercial or financial relationships that could be construed as a potential conflict of interest.
